# Tribo-induced photoluminescent behavior of graphene and YSZ:Er/graphene composite films

**DOI:** 10.1039/c7ra09134f

**Published:** 2018-01-04

**Authors:** Hongyan Wu, Ke Huang, Jianliang Li, Fan Jiang, Xingming Zhao, Lu Wang, Shan Jiang

**Affiliations:** Jiangsu Key Laboratory for Optoelectronic Detection of Atmosphere and Ocean, School of Physics & Optoelectronic Engineering, Nanjing University of Information Science & Technology Nanjing China wuhy2009@nuist.edu.cn 13776500058@163.com jiangfan1109@163.com 13260815003@163.com; College of Material Science and Technology, Nanjing University of Science and Technology Nanjing China jianliangli@163.com; Department of Mechanical Engineering, University of Mississippi, University MS 38677 USA jiang@olemiss.edu

## Abstract

In the present work, a novel method was developed to study the evolving surface state of graphene film as it is subject to friction, characterized by photoluminescence properties. We prepared the graphene film (GF) and YSZ:Er (Er^3+^-Y^3+^ co-doped ZrO_2_)/graphene composite films (ZGCF). The Raman spectra and photoluminescence properties of the GF and ZGCF were characterized before and after the sliding friction. A remarkable phenomenon was observed that after friction the GF generated a more pronounced luminescence response than it had prior, apparently due to graphene quantum dots which were found in the wear debris of the GF. Furthermore, the introduction of graphene into YSZ:Er nanoparticles (NPs) resulted in an unmistakable red-shift on the main luminescence bands of ZGCF after the applied friction. This is explained by the formation of considerable graphene scrolls in the wear debris of ZGCF due to the interaction of the graphene and the YSZ:Er NPs. It can be concluded that changes to the configuration of graphene greatly influence the tribo-induced photoluminescence response. Our findings justify further investigation into the composition and morphology of worn surfaces in order to better understand how photoluminescence relates to frictional effects. In addition, this work proposes the *in situ* fabrication of graphene quantum dots and nanoscale scrolls as a new potential application of the tribo-induced photoluminescence study.

## Abstract 2

In the present work, a novel method was developed to study the evolving surface state of graphene film as it is subject to friction, characterized by photoluminescence properties.

## Graphical abstract

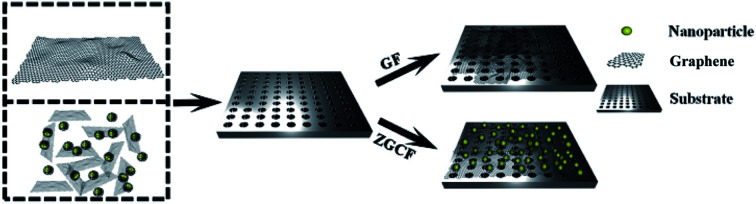

## Background

Extreme external loading conditions can significantly impact the worn surfaces of many materials that are widely used in industrial and marine fields.^1,2^ However, attempts to observe and predict those changes at nanoscale or microscale levels proved to be especially difficult.^3,4^ Therefore, a deeper understanding of the friction process is sought in order to develop an effective early warning system capable of monitoring and locating friction-induced failure *in situ*. In the past decade, several tribological mechanisms have been proposed to explain the friction processing of freshly worn surfaces along with the associated formation and transfer of debris.^5,6^ However, due to limitations of the experimental tools, it remains challenging to thoroughly analyze the role of shearing reactions during the friction process. Of particular interest is the shearing phenomena within the sliding contact area *in situ* at the molecular-level.^7^ Certain weak physical and chemical effects observed on the surface or the interface of sliding partners were typically neglected in the past, but presently these phenomena become increasingly significant in regard to changing friction service conditions.^8^ For example, harsh friction conditions can lead to a cascade of excitation and de-excitation processes of tribological interface or fractured surfaces, producing luminescence and emitting charged particles. Consequently, several investigations on tribo-induced magnetization,^9^ luminescence^10^ and surface charges^11^ have been reported. Nakayama *et al.*^12,13^ studied the phenomena of triboplasma and triboemission, as well as the occurrence of shearing reactions during the friction process. They found that various solids, especially insulating solids, emit UV photons during the sliding process; they also found that the triboemission depends greatly on the electric resistivity of the sliding partners. Nevshupa^14^ found that energy dissipation at a tribo-contact can depend on a variety of elementary physical processes and chemical reactions; they studied several factors, such as the surrounding gas, the surface coating and the types of rubbed materials, that can influence triboluminescent behavior.

Graphene, as a 2D-honeycomb structural carbon sheet, possesses exceptional optical, electrical and mechanical properties.^15–17^ Recently, due to its compact size and extremely thin laminated structure, the tribological properties of graphene have been intensively examined for potential applications in micro- and nano-mechanical devices.^18,19^ The size effect of graphene in conjunction with a surface coating of functional groups or metallic ions was proved to contribute to extraordinary optical properties.^20,21^ It is also known that friction may induce considerable changes in the size, defect structure, and surface adsorption of counterface materials.^22^ Mishina *et al.*^23^ reported an *in situ* observation of such effects on surface crystal grains during a sliding friction process and witnessed a wear debris generated from the solid surface. However, up to now, research has been rarely focused on how the morphologies of graphene wear-debris could affect the physical phenomena and chemical reactions on the tribological surfaces or the graphene interface, in particular regarding the tribo-induced photoluminescence during the process of sliding friction.

**Fig. 1:**
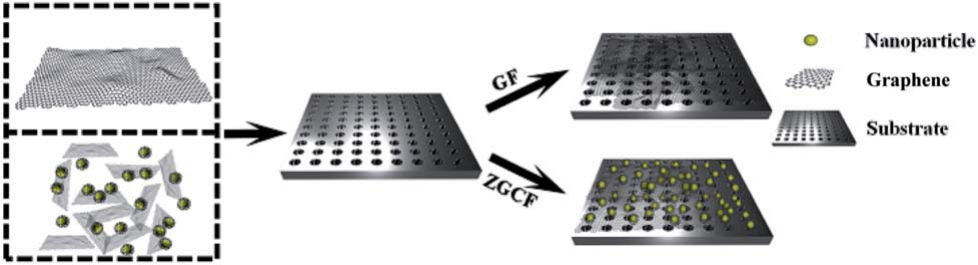
The schematic diagrams of the GF and ZGCF composition films.

In the present work, we prepared graphene film (GF) and YSZ:Er/graphene composite film (ZGCF) (see Fig. 1) and found that both of them possess excellent friction-proof and wear-proof properties. A new method was proposed to probe the properties of both surface and interface of the sliding partners during the friction process. In addition, the wear-debris composition and the surface defects of GF and ZGCF were investigated both before and after friction, with the purpose of analyzing the tribo-induced surface state and the interface phenomena in the counterface materials. Here, we also report a remarkable phenomenon that the GF generated a more pronounced luminescent response after friction than it had prior, due to the graphene quantum dots found in the wear debris of the GF. The corresponding photoluminescence and tribological mechanisms were also discussed in this work.

## Experimental methods

### Laser surface texture of substrate and films

The surfaces of stainless steels (20 mm × 20 mm × 3 mm) were mechanically polished *via* a standard metallographic procedure and then were cleaned with acetone. Subsequently, the treated stainless steels were implemented using the laser drilling technique with pulsed laser (ND:YAG, 20 kHz). The diameter of each hole was 150 μm and each two of the adjacent rows was evenly spaced with 350 μm.

### Synthesis methods of GF

Graphene oxide was synthesized by the Hummers method. The first step consists of the oxidation of graphite using KMnO_4_ as an oxidizing agent. For this purpose, 50 mL of H_2_SO_4_ (AR) was introduced into a three mouth flask with constant stirring. 1 g flake graphite (99%, mesh: 80) and 1.2 g KNO_3_ (AR) were added into the three mouth flask. After stirring for five minutes, 6 g KMnO_4_ (AR) was slowly added to the mixture. The reaction mixture was maintained at 40 °C for 3 h. Then, 80 mL deionized water was slowly added into the three mouth flask and the mixture was maintained at 70 °C for 30 min. After the oxidation step, the resulting mixture was added to a beaker containing 400 mL of deionized water and 20 mL of H_2_O_2_ (GR, 30 wt%) in order to quench the reaction. 2 mol L^−1^ NaOH (AR) solution was slowly added into the above-mentioned solution in order to adjust the PH value to 6. After static precipitation and removing the supernatant, the precipitate was centrifuged and washed with distilled water and ethanol for three times. The product obtained was reduced by hydrazine hydrate (AR, 80%). The obtained reduced graphene oxide (rGO) obtained *via* Hummers' method was dispersed in ethanol at a pre-determined ratio using the ultrasonic treatment. A spin-coating on the stainless steel was applied and dried in an oven at 80 °C. The preparation process of the GF is shown in Fig. 1.

### Synthesis methods of ZGCF

YSZ:Er (Er-doped yttria stabilized zirconia (YSZ)) nanoparticles (NPs) were prepared by the hydrothermal synthesis method. YSZ:Er was prepared by a hydrothermal method. ZrOCl_2_·8H_2_O (AR), ErCl_3_ (99.9%), Y(NO_3_)_3_·6H_2_O (≥99.99%), NaOH (AR), lactic acid (AR) and deionized water were used as raw materials. A procedure for the sample with 1 at% Er^3+^, 10 at% Y^3+^:ZrO_2_ synthesis is typically described as follows: 2.578 g ZrOCl_2_·8H_2_O, 0.3064 g Y(NO_3_)_3_·6H_2_O (acted as stable agent), 0.0219 g ErCl_3_ were first dissolved in 24 mL deionized water. Then 5 mL lactic acid was added to the above solution then mixed well with a magnetic stirrer. 10 mol L^−1^ NaOH solution was slowly added into the above solution to adjust the PH value to 6. After mixing evenly, the mixed solution was transferred to Teflon liner. Hydrothermal treatments were conducted at 160 °C for 24 h. After the synthesis reaction, the product obtained was centrifuged and washed with distilled water and ethanol, and dried at 80 °C during 12 h. In the last step, the dried YSZ:Er samples were annealed at 700 °C for 2 h in air. YSZ:Er NPs, rGO obtained *via* Hummers' method, and salicylic acid were dispersed in ethanol at a pre-determined ratio using the ultrasonic treatment. A spin-coating on the stainless steel was applied and dried in an oven at 80 °C. The preparation process of the ZGCF is also illustrated in Fig. 1.

### Characterization

The dry friction test at room temperature in air was conducted with a WTM-2E tribometer (Lanzhou, China) in a ball-on-disc contact configuration at a load of 1 N and a speed of 200 rpm for 20 minutes. Commercially available silicon nitride balls (Si_3_N_4_) with diameter of 3 mm, and announced mean roughness of 0.02 μm were used as the stationary upper counterparts. The friction-coefficient-*versus*-time curves were generated automatically. All experiments were performed under ambient conditions of 25 °C and 26% relative humidity.

To study the friction effects on the configuration of GF and ZGCF, the morphology and structure of the wear debris were characterized by using high resolution transmission electron microscopy (HR-TEM, Tecnai F20 FEI).

Raman and photoluminescence spectroscopy were performed in the wear and non-wear track by using the laser spectrometer (Horiba Jobin Yvon, Labram HR800) with 514 nm and 325 nm excitation wavelengths to assess the quality of graphene and the luminescent behavior before and after friction. The light source with excitation wavelength was used to test each sample at three random points.

## Results and discussion

**Fig. 2:**
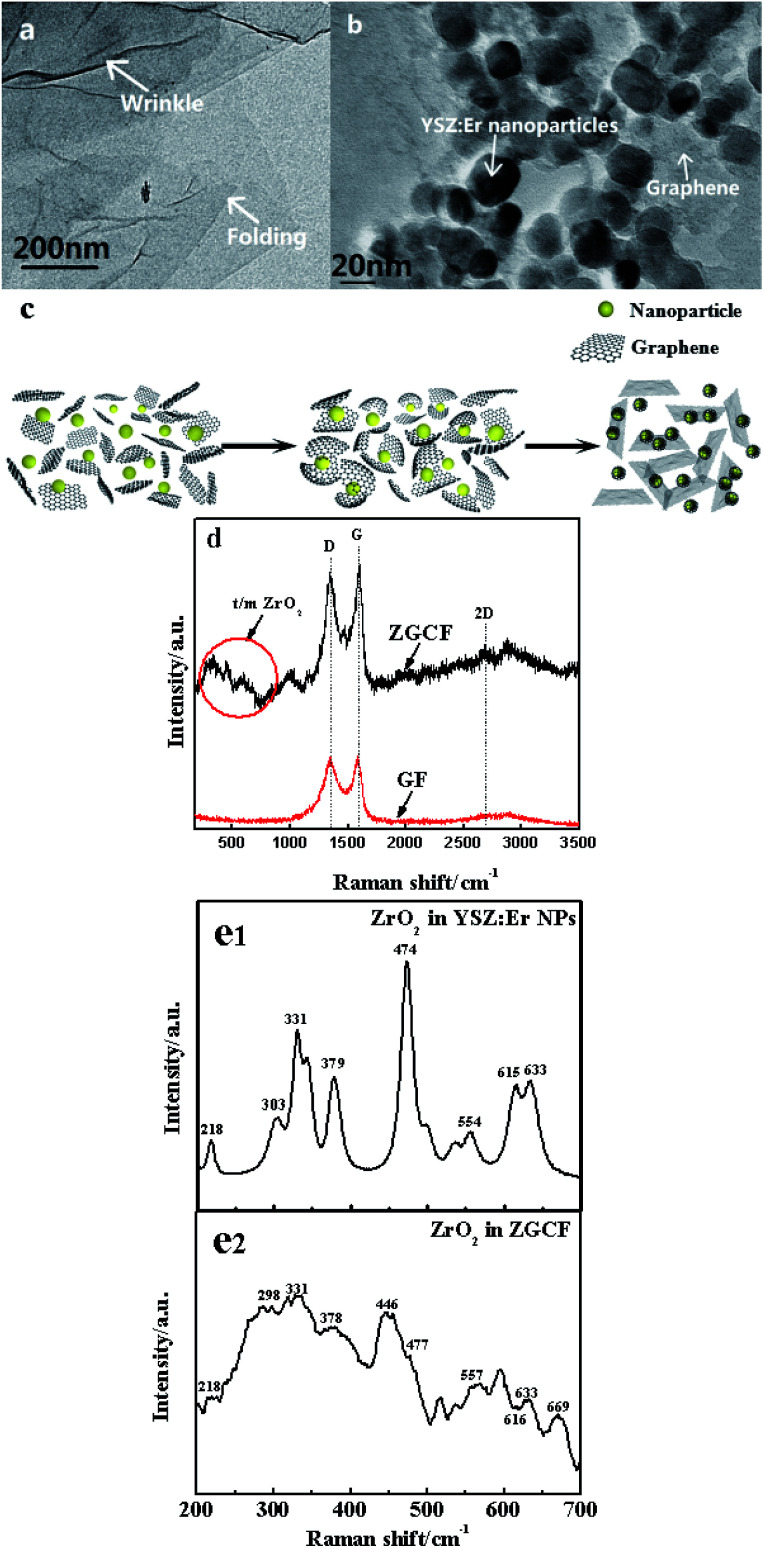
HR-TEM morphologies of (a) graphene. (b) YSZ:Er NPs wrapped in the graphene. (c) The schematic diagram of graphene sheets wrapping YSZ:Er NPs. (d) Raman spectra of graphene and YSZ:Er/graphene nanocomposite. Raman spectra of (e1) ZrO_2_ in YSZ:Er NPs and (e2) that in ZGCF marked by the red circle in (c), respectively.

Fig. 2 shows TEM and Raman spectra with 514 nm excitation wavelength for the GF and ZGCF before the friction tests. Fig. 2(a) shows the folding and wrinkles in graphene. The wrinkling refers to a local folding that forms wrinkles on a flat graphene sheet. The instable folding and wrinkles may be due to the non-uniform thermal expansion in graphene sheets, which facilitate the aggregation and embedding of nanoparticles. Fig. 2(b) shows the HR-TEM image of the formed YSZ:Er-graphene nanocomposite. The size of YSZ:Er NPs (20–50 nm in diameter) in an aqueous suspension were directly added to a dispersion of micrometer-sized graphene sheets, shown in the schematic diagram of the evolutions of graphene wrapping NPs in Fig. 2(c), which is consistent with the TEM analysis. The results display the successful embedding of YSZ:Er into the graphene nanosheets matrix and illustrate that the NPs induced the scrolls of graphene sheets and in turn affected the size, shape and configuration of graphene sheets. These NPs were anchored on the graphene supports due to the strong electrostatic interactions between the oppositely charged graphene sheets and YSZ:Er NPs. Fig. 2(d) shows typical Raman spectra (*λ*_ex_ = 633 nm) of graphene and YSZ:Er/graphene nanocomposite. On the Raman spectrum of graphene, the exhibited G band (∼1605 cm^−1^) is corresponding to sp^2^-hybridized carbon while a D band (∼1350 cm^−1^) is corresponding to disordered carbon. The spectra of YSZ:Er/graphene shows a combination of the characteristic peaks from both the graphene sheets (*i.e.*, the G band at 1598 cm^−1^ and D band at 1344 cm^−1^) and the ZrO_2_ (m-phase at A_g_ 634 cm^−1^ and t-phase at E_g_ 269 cm^−1^ and B_1g_ 312 cm^−1^). The measured signal intensity of D and G peaks of the ZGCF sample is higher than that of GF under the same testing condition. The difference in the Raman spectra of GF and ZGCF is due to the surface enhanced Raman spectroscopy (SERS) effect caused by chemical enhancement. It is generally known that there are many functional groups on the surface of GO, which can interact with YSZ:Er NPs. The obtained ZGCF is something like charge transfer complex, which can absorb the light at the excitation frequency, consequently resulting in chemical SERS.^24^Fig. 2(e1) and (e2) show the Raman spectra of the ZrO_2_ in YSZ:Er NPs and ZGCF, respectively, which indicate that the characteristic peaks of both ZrO_2_ in YSZ:Er NPs and ZGCF are similar. The difference in the peak intensity of is attributed to the presence of graphene wrapping NPs in ZGCF, which is caused by the interaction between the graphene and YSZ:Er NPs.

**Fig. 3:**
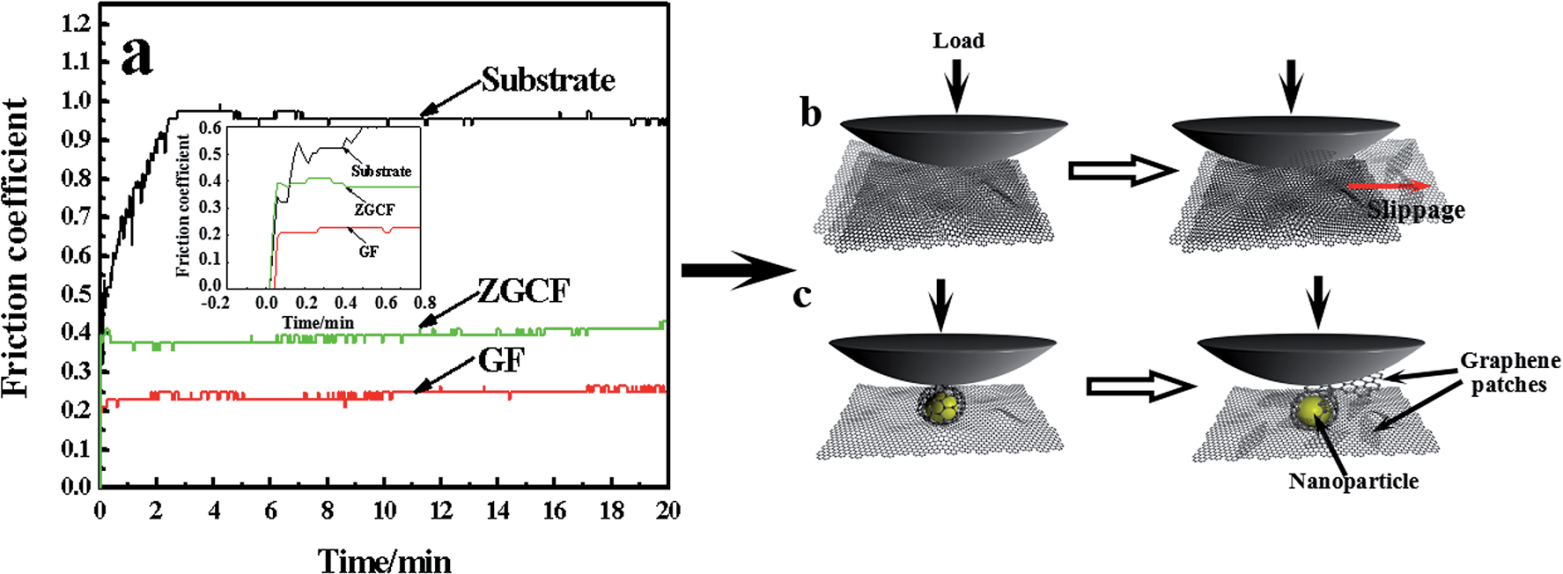
(a) The coefficient of friction (COF) *vs.* time in substrate, GF and ZGCF, and the schematic diagrams of (b) GF and (c) ZGCF during friction process.

Fig. 3 shows the coefficients of friction (COFs) of substrate, GF and ZGCF and the contact schematic diagram of the frictional couples. The comparison of COFs of substrate, GF and ZGCF is displayed in Fig. 3(a). At the initial stage of friction, the COFs of GF and ZGCF are more stable than that of stainless steel (see inset of Fig. 3(a)). After the friction coupling stage, the GF has an average COF of 0.23, while the COF of ZGCF is approximately 0.38. Both of them are much lower than that of the substrate (0.98), which indicates that the introduction of graphene decreases the COF of ZGCF. The frictional process of GF and ZGCF can be analyzed by the contact schematic diagrams of the frictional pairs, as shown in Fig. 3(b) and (c). Fig. 3(b) showed the frictional coupling between GF and the counterpart. The friction action of GF mainly occurs on graphene and Si_3_N_4_ counterface. Owing to the fact that the graphene sheets exhibit a relatively low shear strength, the interlayer interaction is weakened and thus deceases the COF. In contrast, Fig. 3(c) indicated that the friction action of ZGCF mainly occurs on the YSZ:Er (wrapped by graphene) and Si_3_N_4_ counterface. The wrapping around the YSZ:Er NPs by graphene starts at the initial friction stage,^25^ which coincides with substantial dropping of the COF value. As the friction proceeds, graphene sheets are divided into a considerable number of patches and then the YSZ:Er NPs are exposed on the wear surface. Hence, the YSZ:Er NPs act as a nano-ball bearing and can roll between the Si_3_N_4_ counterface and substrate, which reduces the friction and provides extra mechanical strength. Therefore, the COF of ZGCF has a higher COF than that of GF, while both of them have the lower COF than the substrate.

**Fig. 4:**
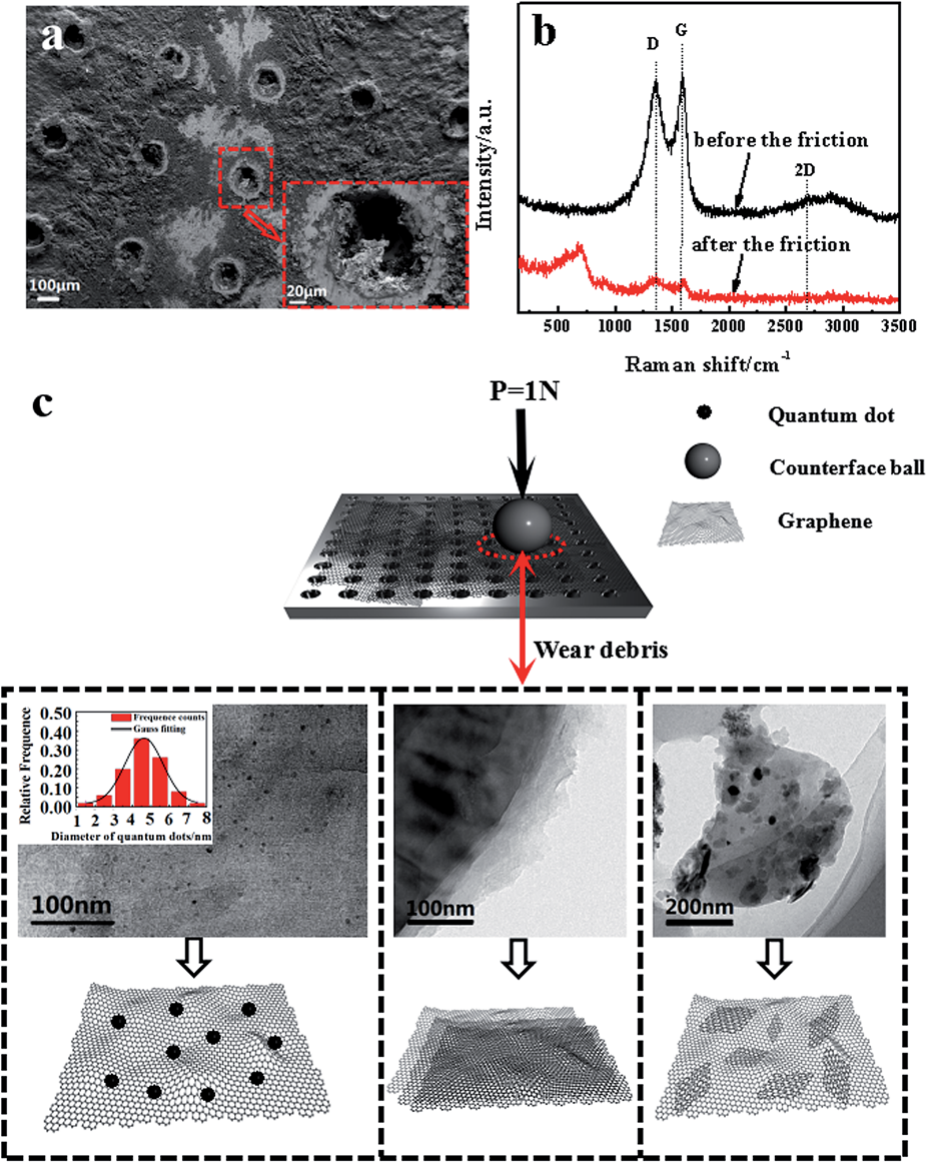
(a) Scanning electron microscopy (SEM) images of the worn surface morphologies of GF after the friction. (b) Raman spectra of GF before and after the friction. (c) TEM of the wear debris of GF and the evolution sketches of the wear debris after the friction.

Fig. 4(a) and (b) show the SEM images of the worn surface morphologies and Raman spectra with 514 nm excitation wavelength of the GF before and after friction respectively. It is observed that no significant substrate wearing/scratching occurs and a continuous compact film is formed on the wear track; this phenomenon indicates that the adhesive wear is obviously caused by the plastic deformation. The continuous compact film on the wear track is the composition of the mixture of graphene and oxidation debris. It can be proved by Raman spectra, which displayed the D band and G band of graphene oxide and the peak value of 667 cm^−1^ of iron oxide debris besides in Raman spectrum of GF after the friction. As for the new band peak located at 667 cm^−1^ of GF, many researches have reported^26–28^ that the peaks at about 660 cm^−1^ exhibited existence of the magnetite (Fe_3_O_4_). The hole structure produced by laser drilling can not be filled by the graphene sheets, which affects the release of the graphene lubricant and the wear life of GF. The Raman spectra show that the intensity ratio of D band over G band (*I*_D_/*I*_G_) after the friction is higher (1.03) than that before friction (0.95), as indicated in Fig. 4(b). This result is consistent with previous research,^29^ where the increase of *I*_D_/*I*_G_ ratio is due to the increased number and size of sp^2^ clusters. The surface structure and composition of graphene sheets were greatly shown to be influenced by plastic deformation and heat transfer during the friction; these two factors play an important role in the evolution of graphene configurations.^30,31^ The insets of Fig. 4(c) show schematic illustrations of the wear debris in GF before and after the friction sliding. Due to the frictional effect the size of graphene decreases and graphene patches are formed. Most notably, a substantial number of graphene quantum dots is found in the wear debris on the surface of GF and the average size of the quantum dots is about 4.58 nm, shown in the inset of Fig. 4(c). Simultaneously, the defect-like structures is observed after the abrasive process, which considerably affect the luminescent behavior of the graphene.

**Fig. 5:**
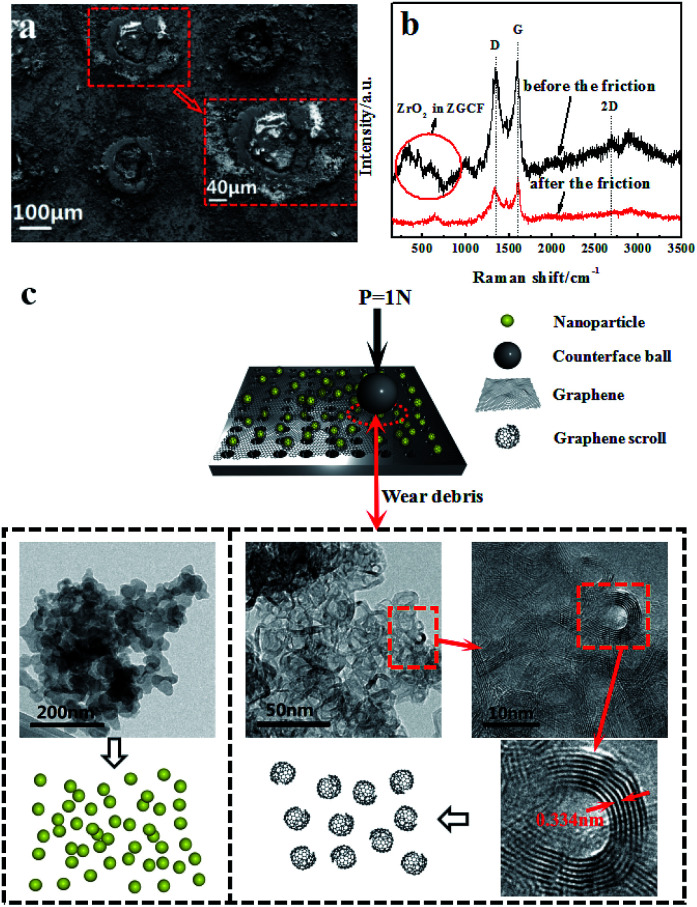
(a) SEM images of the worn surface morphologies of ZGCF after the friction. (b) Raman spectra of ZGCF before and after the friction. (c) TEM of the wear debris of ZGCF and the evolution sketches of the wear debris after the friction.

Fig. 5(a) shows the SEM images of the worn surface morphologies of the ZGCF. It is observed that evident abrasive/adhesive wear forms on the surface of the ZGCF, and this adhesive wear occurs around the laser-drilled holes that are filled with NPs. The graphene sheets first wrap around the NPs, but as the friction proceeds the curled graphene sheets is destroyed. Thus, the friction action is mainly between the NPs and grinding ball, and the increase of COF (which is discussed in the above-mentioned analysis of Fig. 3) implies that NPs have an important role in bearing the friction load. Therefore, the ZGCF have a potential application in improving the wear life of graphene sheets. Fig. 5(b) shows Raman spectra (with 514 nm excitation wavelength) of the ZGCF before and after friction. It can be found that the Raman band of ZrO_2_ shifts from 634 cm^−1^ (before friction sliding) into 642 cm^−1^ (after friction sliding). Interestingly, unlike the case of GF, the *I*_D_/*I*_G_ before friction (0.97) is surprisingly higher than that after friction (0.83), indicating that the embedded YSZ:Er NPs do affect the defect structure in the graphene. Specifically, when these YSZ:Er NPs are removed from the graphene, we observed the formation of many graphene scrolls that are very similar to CNT-like structures^32^ (see the TEM of wear debris in Fig. 5(c)). To the best of our knowledge, this phenomenon, associated with the shape and size of NPs as well as the wear debris formed after friction, has not been reported in the past. A previous study^33^ showed that the formation of graphene wrinkles depends on the NPs size and the distance between two neighbouring NPs. This NPs-induced wrinkles can promote the formation of graphene scrolls. Fig. 5(c) shows that a lot of graphene scrolls are inter-connected together. With the participation of YSZ:Er NPs, these formed graphene scrolls are also similar to the disordered carbon shell observed previously.^34^ The formation of the graphene scrolls can be explained by the external loads (sliding frictions) from the encapsulated YSZ:Er NPs that essentially drive the motion of the rolling graphene to form into a scroll shape.^35,36^ During the friction, the shear stress arises and weakens the interaction between the graphene and NPs. When the wear debris is dispersed into an alcohol solution for microstructure characterization, many YSZ:Er NPs become detached from the curled graphene and the hollow graphene scrolls finally can be formed.

**Fig. 6:**
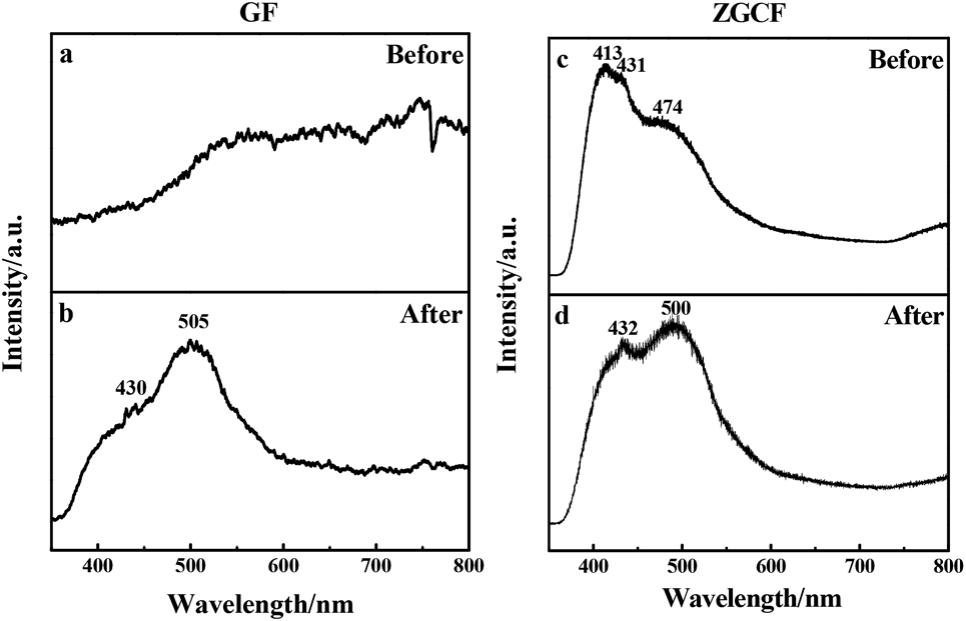
Photoluminescence spectra of (a, b) GF and (c, d) GZCF before and after the friction, respectively.

**Fig. 7:**
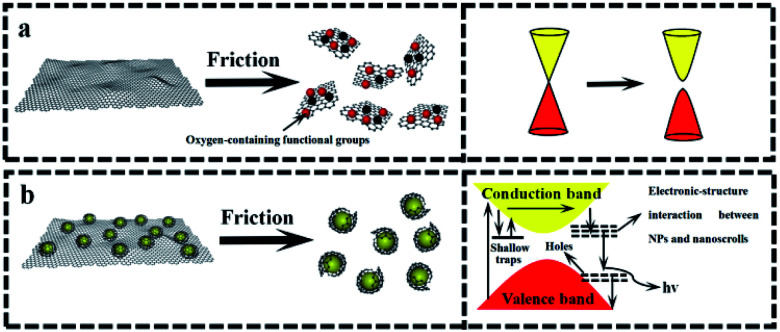
Photoluminescent mechanisms of (a) GF and (b) ZGCF before and after the friction.

The above-mentioned wear debris of unique microstructures culminated in an unexpected physical response, *i.e.*, the tribo-induced photoluminescence. Fig. 6 shows the photoluminescence spectra of both GF and GZCF before and after friction. As can be seen in Fig. 6(a), no evident luminescent signal is found before friction, indicating that regular graphene oxide sheets do not exhibit luminescent capability. As the friction proceeds, there is a shear effect on both the surface size and the defects of GF. In previous work^37,38^ it was found that optical responses are due to the quantum confinement effect of graphene as well as the presence of defects. Consistently, in the present work this obvious photoluminescence behavior of GF is observed at the wavelengths of 430 nm and 505 nm within the spectrum, as plotted in Fig. 6(b). Likewise, the photoluminescence spectra of ZGCF before and after friction are also different, as shown in Fig. 6(c) and (d). In fact, the presence of graphene scrolls and deformation of NPs introduce a change in the spectra characterization, leading to red shifts in the emission spectra of the ZGCF. This finding is consistent with the report in the literature^39^ that graphene-oxide NPs can exhibit a red-shift of the band-edge and a significant reduction of the band gap. These phenomena can be explained by the photoluminescent mechanisms illustrated in Fig. 7. For the friction cases of GF, large-scale graphene sheets with zero bandgap are not easy to produce electron transition and thus cannot exhibit a luminescent behavior. During the friction process, the size of the graphene sheet decreases due to the frictional shear, and a strong local and edge quantum effects can be present, which results in a significant luminescent behavior.^40^ Meanwhile, the friction environment enables the adsorption of a little amount of oxygen on the graphene surface accompanied by the composition of the debris, which gives rise to the presence of sp^3^ carbon atoms covalently bonded to the oxygen-bearing functional groups in addition to the sp^2^ hybridization in the pristine graphene. Therefore, a finite electronic bandgap generated by the disruption of π-networks, to a certain extent, can facilitate in opening up the bandgap of graphene and thus enhance its photoluminescence quantum yield. In contrast, the changing photoluminescent behavior of ZGCF can be explained with a mechanism (see the illustration in Fig. 7(b)) that involves combined effects of both the YSZ:Er NPs and the graphene scrolls during the friction process. The YSZ:Er NPs covert the graphene from the lamellar structure into a scroll configuration that also in a nanoscale size comparable with the NPs. The formation of nanoscrolls also displays the local boundary effect that is similar to the graphene quantum dots, and resulting in a change of the photoluminescence behavior. Additionally, the non-centrosymmetric nature of these YSZ:Er NPs can also induce a piezoelectric effect. As illustrated in Fig. 7(b), this leads to a decrease of the trap-depth and can cause the detrapping of electrons from filled-electron traps, which enables the electrons to reach the conduction band. The electrons may recombine with the holes trapped in the defect centres or they may fall into the valence band with the energy that is released non-radiatively.^41^ Our current study demonstrates that the tribo-induced interaction between YSZ:Er NPs and graphene nanoscrolls has a unneglectable effect on the electronic structure and bandgap, which in turn influences the luminescent emission and results in the red-shifts on the spectra.

## Conclusions

In summary, we proposed and implemented a novel strategy for studying the surface state and interface phenomena of sliding contact materials during the friction process. More importantly, we report the phenomenon that GF exhibits pronounced friction-induced photoluminescent properties due to the formation of graphene quantum dots as well as the defect structures formed in the graphene sheets. Furthermore, ZGCF displayed a noticeable red-shift of luminescent bands due to the combined effects of YSZ:Er NPs and graphene scrolls during the friction process. The discovery of the presence of graphene quantum dots and graphene scrolls that are induced by friction provides a potential new approach for multi-morphology fabrication of graphene. Lastly, the tribo-induced luminescent behavior reported in current research can be served as an incentive for further inquiry into tribo-physical phenomena and tribo-chemical reactions in the near future.

## Conflicts of interest

There are no conflicts to declare.

## Supplementary Material
